# “Not Sure Sharing Does Anything Extra for Me”: Understanding How People with Cardiovascular Disease Conceptualize Sharing Personal Health Data with Peers

**DOI:** 10.3390/ijerph19159508

**Published:** 2022-08-02

**Authors:** Jhon Adrián Cerón-Guzmán, Daniel Tetteroo, Jun Hu, Panos Markopoulos

**Affiliations:** Department of Industrial Design, Eindhoven University of Technology, 5612 AE Eindhoven, The Netherlands; d.tetteroo@tue.nl (D.T.); j.hu@tue.nl (J.H.); p.markopoulos@tue.nl (P.M.)

**Keywords:** cardiovascular disease, data sharing, peers, personal health data, qualitative survey data, thematic analysis

## Abstract

As people deal with cardiovascular disease (CVD), they are to self-monitor routinely and be aware of complications and the corresponding course of action. Engaging in these self-care behaviors is conducive to gaining knowledge of health status. Even so, knowledge of the self may be insufficient in making sense of chronic conditions. In constructing a new normal after health-related life disruptions, people often turn to peers (others facing similar health issues) and share personal health information with each other. Although health information-sharing behavior is well-documented, it remains underexplored what attitudes individuals with chronic conditions, such as CVD, have toward disclosing personal health data to peers and exploring those of others with similar conditions. We surveyed 39 people who reported being diagnosed with CVD to understand how they conceptualize sharing personal health data with their peers. By analyzing qualitative survey data thematically, we found that respondents expressed themselves as uncertain about the benefits of interacting with peers in such a manner. At the same time, they recognized an opportunity to learn new ideas to enhance CVD self-care in mutual data sharing. We also report participants’ analytical orientation toward this sort of data sharing herein and elaborate on what sharing a range of personal health data could mean. In light of the existing literature, this study unpacks the notion of sharing in a different population/pathology and with more nuance, particularly by distinguishing between disclosing one’s data and exploring others’.

## 1. Introduction

### 1.1. Background

People with cardiovascular disease (CVD), and their informal caregivers, are primarily responsible for self-care. Patient encounters, or interactions between individuals and healthcare providers, have been estimated to add up to only 10 hours per year on average [1]. Under these circumstances, people need to engage in health maintenance, monitoring, and management activities—the core elements of self-care [1]—to exercise control over their own health. Here, gaining knowledge of health status is an essential individual-level self-care behavior. In order for knowing their health status to be beneficial, people are to self-monitor routinely and, notably, be aware of risks of complications and the corresponding course of action [1].

Nonetheless, the clinical literature has reported that people with heart failure, a CVD condition, lacked skills in response to symptoms (i.e., self-care management) [1]. The time between the onset of worsening disease and healthcare utilization for them was often in the order of days, where immediate action would have been beneficial. The information science literature, for its part, showed that knowledge of the self could be insufficient in making sense of symptoms, their triggers, and treatments of chronic conditions [2].

As people deal with chronic conditions, they turn to their peers (others facing similar health issues) to validate personal experiences [2]. Thus, the experiences of similar others often shape individuals’ notions of “normal”—meaning socially constructing a sense of order amidst the chaos [3] (as cited in [4]). In constructing a new normal after health-related life disruptions, people devote a great deal of effort to information behaviors [4], including sharing personal health information—from physiological data to self-care strategies, challenges, and experiences (e.g., ref. [5]).

The literature has addressed health information-sharing behavior by adopting predominantly quantitative approaches to research (e.g., refs. [5,6,7]). In contrast, works grounded in qualitative orientations (e.g., refs. [8,9]) are somewhat scarce [10]. The contexts for this sharing are between patients and healthcare providers (e.g., ref. [9]) and among peers (e.g., refs. [5,6,7,8]), to name a few. Sometimes, sharing personal health information is discussed under similar terms, such as “disclosure” (e.g., ref. [7]). At the expense of oversimplification, the existing research has primarily focused on what health information people are likely to share and what factors influence their willingness to do so. In motivating their work, scholars point out presumable benefits that engaging in data sharing yields for individuals. Among them are increased social support (e.g., refs. [5,6,10]) and enhancing self-care decision making (e.g., ref. [10]).

However, how people with chronic conditions actually feel about sharing their health data with their peers is underexplored [8]. Indeed, a recent narrative review called for more qualitative research on this topic [10]. For this reason, we argue that investigating people’s attitudes toward disclosing personal health data to peers and exploring those of others in similar positions emerges as an exciting topic of inquiry.

To our knowledge, Bussone et al. [8] is the only earlier study that, adopting a qualitative orientation, focuses on the sharing of personal health data among peers. Theirs explored the trust, identity, privacy, and security concerns people with the Human Immunodeficiency Virus (HIV) had in data sharing. In doing so, they drew design considerations to facilitate sharing personal health data in an online health community, including giving individuals control over what and with whom to share and encouraging appropriate, objective, and balanced data sharing. Still, a broader perspective on how people conceptualize the sharing itself, rather than particular considerations for doing so, is lacking. Moreover, hearing the voices of the “non-aligned” could enrich this perspective, in contrast to the sample in Bussone and colleagues’ study, which was composed solely of people interested in sharing their health data with their peers.

### 1.2. Objective

The contribution of this study is to provide an understanding of how people diagnosed with CVD conceptualize sharing personal health data with peers. We identify and develop three patterns of meaning around this sort of data sharing, together with what it could mean to share a range of personal health data. Additionally, we suggest two design opportunities for collaborative health technology through the sharing of personal health data among peers.

## 2. Materials and Methods

### 2.1. Study Design

**Figure 1:**
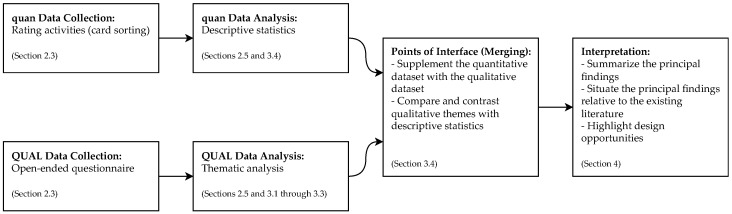
Diagram of the convergent design of the study presented herein, following the notation system for drawing diagrams of design [11]. The quantitative data collection and analysis appear at the top of the figure, while the qualitative aspects appear at the bottom. We implemented the quantitative and qualitative strands simultaneously but placed greater emphasis on the latter within the study, as indicated by using lowercase (“quan”) and uppercase (“QUAL”) letters. The figure also shows the merging of the two strands and the overall interpretation.

This research study was grounded in a convergent mixed methods design [11]. Figure 1 depicts the flow of the quantitative and qualitative strands and the procedures for mixing them. In this design, we simultaneously collected quantitative (ratings, numeric-like) and qualitative (text) data through a predominantly qualitative survey [12]. The survey contained a number of open-ended questions to elicit attitudes toward disclosing one’s data and exploring others’, along with rating activities to learn what pieces of their personal health data participants were likely to share with peers. We then analyzed both datasets separately, but with the help of the qualitative data, we elaborated on what the quantitative results could mean. Here too, we compared and contrasted the qualitative themes with descriptive statistics.

The primary objective of this research is to understand how people with CVD conceptualize sharing personal health data with peers—which is why we placed greater emphasis on the qualitative strand within the study. However, gauging how participants’ conceptualization(s) were reflected in the data items they chose to share, we believed, would lead us to points of convergence and divergence between participant views and ratings, which in turn would enhance our understanding of the topic of inquiry. The above explains our choice of a convergent mixed methods design.

In this study, we not only used qualitative data collection and analysis techniques but also implemented them within a qualitative paradigm. Hence, we grounded our qualitative research design in a “Big Q” approach [13,14]. In “small q” qualitative research, the researcher addresses positivist–empiricist research concerns such as impartial and unbiased knowledge and inter-rater reliability. In contrast, a distinctive element of “Big Q” qualitative research is the recognition of the researcher’s subjectivity in the research process, treating it no longer as a weakness but as a strength [13,14]. Thus, we acknowledge that we were not neutral in searching for patterns in the qualitative data and that our involvement and partiality shaped the qualitative results.

### 2.2. Participants

This research study followed a self-selection recruitment strategy. Hence, participants, aged 18 or above and fluent in English, were people who self-reported having been diagnosed with CVD by a medical doctor, particularly with coronary artery disease (CAD) or heart failure (HF). We chose to recruit people with CAD or HF since they are to engage in self-monitoring routinely and are to (gain knowledge to) be aware of symptoms of worsening disease and the corresponding course of action [1].

In total, we reached out to 63 potential participants through Prolific (www.prolific.co, accessed on 27 June 2022)—an online platform connecting researchers with their target audience by enabling them to apply a host of prescreening filters. Five of the potentially eligible participants did not consent to participate, 14 did not meet the eligibility criteria, four dropped out, and one did not follow the study instructions. Therefore, our final sample consisted of 39 participants who completed the survey and whose responses constitute the dataset this paper analyzes. We validated participants’ prescreening response (having self-reported being diagnosed with CAD or HF by a medical doctor) at the beginning of the survey. For this reason, those who provided conflicting information were excluded from the survey.

Among the final sample, 46.2% (18) and 51.3% (20) of participants identified as female and male, respectively; one as non-binary. More than half (21, 53.8%) were aged 45 years or older. Geographically speaking, they resided mostly in the United Kingdom (10, 25.64%), South Africa (8, 20.51%), Poland (7, 17.95%), Portugal (4, 10.26%), and the United States (3, 7.69%). With regard to their health condition, approximately equal numbers reported living with CAD (19, 48.7%) or HF (20, 51.3%). Likewise, 69.2% (27) of participants stated they were diagnosed with CVD five years ago or more, 20.5% (8) between two and five years ago, and 10.3% (4) between one and two years ago. We paid participants GBP 8.77 each, the equivalent in January 2022 to the Dutch hourly minimum wage.

We used Prolific to recruit participants on the grounds of recent empirical evidence and the practicality of the platform for this research study. Eyal et al. [15] showed that Prolific could provide high-quality data relative to participants’ attention, comprehension, and honesty. Their research also challenged reputation (approval rating, or the number of accepted submissions divided by the participant’s total) as sufficient for data quality since its contribution seems negligible compared to not applying it. The latter had practical implications in our research, especially in light of the related literature. Chandler and Paolacci [16] (as cited in [15]) showed that non-naïve participants—that is, those who are able, with relative ease, to recognize and respond to common research tasks, such as attention checks [15]—could impact data quality negatively. Since it appears reasonable to infer that highly reputed participants might be less naïve [15], we decided not to filter by reputation. Instead, we applied prescreening filters to select participants who self-reported to have CAD or HF and were fluent in English.

### 2.3. Materials

We used a survey to gather attitudes toward disclosing one’s and exploring others’ data owing to its potential value for qualitative research and suitability to facilitate the research study presented herein. Braun et al. [12] suggested that this data collection method could provide rich and complex datasets on people’s experiences, perspectives, and practices. Because of its practicalities of online delivery and self-administration, the survey can reach out to geographically dispersed populations and thus hear diverse voices, potentially enriching the understanding of the subject of interest [12]. Moreover, when designed to make respondents feel anonymous, the online survey can encourage participation and the disclosure of sensitive information [12].

Two groups of three participants each pilot-tested the survey. Piloting led to a range of refinements, including rephrasing question wording, eliminating questions judged indistinguishable from the others or not relevant to the research question, moving demographic questions to the end of the survey, and making most open-ended questions optional, as described below.

The final survey contained 30 items. Nevertheless, not all participants were asked the same questions, nor were they required to answer in all cases. To illustrate, having first provided them with our meaning of personal health data (“data about your health status you collect, track, or monitor for yourself … at home or in other everyday settings”), we asked participants whether they had collected such data in the past three months. When answering “yes,” follow-up questions revolved around frequency, devices they had used, and optionally describing their experience. When “no,” participants were asked, but not required, for their reasons. By thus using survey logic and making most open-ended questions optional (11 out of 14), we sought to counteract participant disengagement and fatigue. As for the other 16 items, 13 were closed-ended questions, one was a rating activity, and two were attention checks.

We organized the survey into five sections: health condition (four items); self-monitoring (eight); sensemaking in CVD (five); sharing personal health data with peers (10); and demographic information (three). The co-constructing stories participatory design technique [17] guided the survey design. This technique involves two phases to link memories in related contexts to the design concept. Therefore, to elicit experiences relevant to the subject of interest, participants were first asked about collecting personal health data. Following the theoretical framework of sensemaking in chronic disease self-management [18], we subsequently asked them about previous gaps in their understanding of disease-specific situations, how they analytically engaged with such situations, and whether personal health data triggered and enabled their sensemaking.

Buskermolen and Terken [17] suggested that after having evoked relevant past experiences, participants will be better prepared to provide in-depth feedback and suggestions around the design concept. Nevertheless, the designer first introduces the concept in an envisioned context through a fictional story. In light of the latter, participants were presented with a vignette (hypothetical scenario) wherein a fictional character with CAD installed a mobile application (app) to record behaviors and health measurements. Through this app, the character engaged in peer data sharing to contextualize his health status. We explained our notion of peer and data sharing as follows: “[Your peers are people] living with cardiovascular disease and similar illness experiences. … In this exchange, you can choose what pieces of your personal health data and who to share with, as can your peers with you.” Other practical aspects were also explained (e.g., who could use the app, data security, and who the app providers were). Only then did we gather attitudes toward disclosing data about oneself and exploring those of others, and engaging in discussions with peers about each other’s data.

Lastly, we borrowed inspiration from the card sorting method [19] and Bussone et al. [8] to gauge how participants’ conceptualization(s) reflected in the pieces of personal health data they were likely or unlikely to share with peers. Specifically, participants were tasked with sorting a range of data items into five scale-like categories, from 1—“very unlikely” to 5—“very likely.” Here, we distinguished three types of data (as in Bussone et al. [8]): medical (e.g., diagnosis, medication, and blood pressure), lifestyle (e.g., physical activity, body weight, and diet), and personal (e.g., name, age, and gender).

### 2.4. Procedure

We informed participants that the survey would last about one hour. Since most of the questions were open-ended, we also indicated they were required to elaborate on their views. Upon clicking the survey link, potentially eligible participants learned about the motivation and procedures of the research through the Subject Information Sheet. We suggested that they consented to participate after sufficient reflection. If they agreed to participate and confirmed that they had CAD or HF, we directed them to complete the survey. The survey’s average completion time was 47.45 minutes (SD=25.19).

We used Qualtrics survey software to collect participant responses anonymously. To prevent multiple submissions, we placed a cookie in the participant’s browser to flag survey takers through the survey security options, especially since several hundreds of Prolific users had self-reported CAD and HF. Under this circumstance, we could not rule out any chance of selecting the same participant multiple times. (During data cleaning, we removed the two submissions from the only participant who managed to circumvent the security option.) Only after obtaining approval for the research study from the local Ethical Review Board of the Department of Industrial Design, Eindhoven University of Technology, did we approach participants.

### 2.5. Analysis

This paper analyzes qualitative data following a “Big Q” approach [13,14] to understand participant-defined meaning around sharing personal health data with peers. We used thematic analysis (TA) to identify and interpret patterns of meaning (themes) [20,21]. Our approach to TA was inductive, developing codes and themes from the data. We started familiarizing ourselves with the dataset by reading and re-reading it several times. Then, our coding focused on semantic (descriptive) and latent (interpretative) features of the data content. In coding and subsequently developing themes, we aimed to capture participants’ accounts to the best of our ability while acknowledging that our involvement and partiality shaped the outcome of TA. More precisely, we adopted an experiential orientation within an epistemologically constructivist framework to address the research question [13,21,22,23] (as cited in [14]).

After recursively refining codes, we moved on to generating initial themes by clustering the former around a central organizing concept. In this phase, thematic maps proved useful. The lead author carried out all the coding and initial theme generation. The co-authors engaged in subsequent phases of further developing, reviewing, and finishing themes through discussion and revision to the manuscript. This engagement with the data we have narrated built upon Braun and Clarke’s theoretically flexible TA [20,21], which we chose to inform our analysis since it aims at the exploration of meaning and recognizes the researcher’s subjectivity in the process. Therefore, here we do not report on measures of quality such as inter-rater reliability or coding agreement, which stem from “small q” orientations and are incoherent with reflexive TA [24].

With regards to participants’ ratings—quantitative data—we first linearly transformed them into the range 0–100 using min–max normalization, thereby achieving a more intelligible and standard representation of how likely or unlikely participants were to share a range of pieces of personal health data with peers. Where a participant rated a data item as “1—very unlikely” to share, we transformed it into 0; conversely, where they rated it as “5—very likely,” we transformed into 100. Then, we calculated mean ratings for data items and supplemented descriptive statistics with participants’ reasons for their choice.

## 3. Results

### 3.1. Theme 1: “Not Sure Sharing Does Anything Extra for Me”

In elaborating their view on sharing data with peers, responses reflected somewhat a mental calculation by which participants situated the potential benefits of such sharing relative to existing sources of self-care support. To illustrate, the quote that describes the present theme shows that one respondent (P2, male, 55–64 y/o, CAD, five years or more after diagnosis) could not see any added value in interacting with peers in that way. What sufficed for self-care was knowing he had the data to help himself deal with his condition or, “unless it’s really serious,” mentioning it to the “cardiologist on [the] next visit.” The mental calculation we refer to is similar to the privacy calculus perspective [25] (as cited in [6]). Frost et al. [6] adopted this perspective to understand how users approached information sharing in an online cancer community. Their results suggested that the study sample was pragmatic in choosing what pieces of personal health data to share and who with. Our participants often expressed themselves pragmatically, too.

A prominent source of self-care support was the self. Specifically, participants with a high sense of self-efficacy [26] (as cited in [27]) felt that it was unnecessary to interact with peers through data sharing, either because of presumable mastery of illness-related experiences or preference for dealing with their condition on their own.

I usually prefer to manage [things] myself. [It’s] my problem and I have the solution. … Don’t look anywhere else for info—it’s all a bit personal for me. Nor do I need any contact with other sufferers. … When I go to see the medical team, I want them to tell me all the detail they have—the rest of the time I prefer to try to put it to the back of my mind and just deal with my own condition on my own. (P2)

On the whole for me presonally [*sic*] I have lived with the condition for quite a few years and dont [*sic*] feel it neccessary [*sic*] to interact in that way [sharing data with peers]. (P30, male, 55–64 y/o, CAD, five years or more after diagnosis.)

P2 and P30, and many others, also agreed on trust in medical professionals—the most frequent external source of self-care support noted by participants. However, not in all cases did opinion formation result from the mental calculation participants seemed to do. Sometimes, they did not value looking at their peers’ data. For example: “I don’t think peer data would help me. I would rather consult a professional” (P1, female, 65–74 y/o, CAD, five years or more after diagnosis). The idea contained in these simple but eloquent words was often central to participant discourse. Thus, respondents appeared to give less value to peer data, as the data—either in the form of statistics or raw numbers—would make sense only to whom they belong.

I’m not sure how I would make use of a peers [*sic*] data. I’m not sure beyond knowing we have the same condition if it is helpful or not to know their numbers … if stats would really help others or not since we all have different baselines, I suppose at a certain point though any symptom can become an ’alarm’ symptom so it’s good to be informed of those numbers (for example like a fever or heart rate that is dangerously high), of course I don’t go to the hospital even when my heart rate is above 190 though I am sure for some that [it] is a rate that would send them to seek emergency medical care. So I find it should be up to [the] individual or the individuals [*sic*] doctors when to seek or not to seek medical care. (P25, female, 35–44 y/o, CAD, five years or more after diagnosis.)

A few others went beyond this notion of little value and argued that peer data do more harm than good. This detriment, participants noted, could be in the form of new anxieties or even catastrophic outcomes. For instance, first expressing herself as skeptical about sharing her data with strangers, P20 (female, 25–34 y/o, CAD, five years or more after diagnosis) went on to remark:

I am also a little afraid of my peers [*sic*] data because maybe my data doesnt [*sic*] fit theirs to [*sic*] well which might lead to thoughts that my data is off. Even though it probably fits my body etc. … I might excange [*sic*] data with a person who is close to my attributes. But again—I am not entirely certain I would use this app function at all as it [sharing data with peers] might get me more worried about my own condition in comparison than without. (P20)

It was not only that knowing data from others could introduce anxieties for some, but also that peers “can mislead each other”. The female participant authoring these words (P34, 55–64 y/o, HF, two years or more after diagnosis, but less than five) grounded her distrust on the idiosyncrasies of each condition [2]: “No two diseases are the same, no two life situations are the same. Each case is unique”. Next, she warned of a worst-case scenario for peer advice, noting that if one “were to give advice and the advice was bad, [someone] could die”.

While the above and a couple of other participants showed themselves averse to peer advice on the grounds of catastrophic outcomes and the idiosyncrasies of each condition, another positioned her perspective in light of contemporary debates. Still, she suggested that (discovering) others’ inclinations in such discussions would tell her who might be trustworthy.

It is difficult to find peers whose advice you can take on [*sic*] face value … thinking about this and the polarisation of society into left and right politics; vaxxers and anti-vaxxers; homeopathy or allopathic medicine, one might want an optional profile page where you can indicate some of your ’beliefs’/occupation/education level so I know if I can trust you. This has become important recently. … If someone is an anti-vaxxer, I do not want to take advice from them. (P3, female, 65–74 y/o, CAD, two years or more after diagnosis, but less than five.)

So far, we have related that participants appeared to construct meaning around the subject of interest either by situating data sharing relative to existing sources of CVD self-care support or by weighing potential—mainly disadvantageous outcomes. As a result, some expressed uncertain benefits of interacting with peers in that way. Here seems reasonable to think that the more uncertain participants felt about the benefits, the more reluctant they could be to share personal health data with peers. Under this premise, we clustered three notions of reluctance shared by several others into a subtheme within uncertain benefits.

The first notion did not see “any valid reason for sharing data with anyone outside the medical profession” (P30). “Also I am sceptic—who would know that the other person … is medically versed enough to understand … data form [*sic*] others” (P20). Next to the utility of personal health data in the hands of peers was a sense of discomfort or aversion to exposing one’s data to strangers. For instance, P34 noted: “I don’t like to share my health information, I don’t like to share anything about myself. Nobody else’s business”. While ascribing this aversion to data sensitivity might be plausible, some studies suggested that people seem to downplay such sensitivity and instead “care most about the specific purpose for using their health information” [28] (p. 103222). Additionally, the recipient of the data would be of secondary importance. Consistent with this body of literature, some participants expressed flexibility about reconsidering their reluctance as long as they knew how peers would use their personal health data: “It depends on who has access to the data, and how they will use it” (P28, male, 55–64 y/o, CAD, five years or more after diagnosis). Nevertheless, P34 was adamant (“I don’t change my mind for any reason”).

Closely related to aversion toward disclosing personal health data to strangers was unauthorized third-party access to one’s data. For example, despite indicating that sharing data with peers is “great to understand that you are not alone in situatons [*sic*] like that [living with CVD], and that there’s a [*sic*] plenty of people like you”, P7 (non-binary, 18–24 y/o, HF, five years or more after diagnosis) flatly refused to do so for fear of data breaches. The third and final notion of reluctance thus revolved around data security. Even though we suggested that participants could choose what to share, how, and with whom and that there would be compliance and data protection officers, the same participant rounded off their argument this way: “for hackers it’s not a big deal to steal this data”.

Overall, responses we clustered into this theme suggested that uncertain benefits could often make participants reluctant to share personal health data with peers. However, not in all cases did respondents ascribe reluctance to uncertain benefits.

### 3.2. Theme 2: “Comparing Apples with Apples and Not with Pears”: Affordances and Drivers of Connecting with Similar Others

As people deal with life-disrupting, health-related challenges, further exacerbated by each condition’s idiosyncrasies and complexity, they expend great effort in information-seeking behavior to validate personal experiences [2]. A salient type of information people seek out is peer experiences. Indeed, some scholars have suggested that the experience of others facing similar health challenges shapes individuals’ notions of normal—thereby conceptualizing normal as socially constructed [4]. However, early in the process, people need to address how to find others in similar situations.

Central to participant discourse was often the assertion that sharing personal health data serves to connect people with others who have similar health issues. The second theme thus clustered comments that revolved around connecting with similar others and captured affordances and their drivers. For example: “I like that it [data sharing] helps them [peers] realize that they are not alone and someone out there is also going through the same thing” (P13, female, 25–34 y/o, HF, one year or more after diagnosis, but less than two).

One of the most prominent affordances participants frequently referred to was that of social comparison. This comparison operated to serve several purposes. The first of these was as a source of vicarious experiences.

I think sharing health information can be really helpful. … On the one hand, it motivates me when I compare the results of others [with mine], and on the other hand, others can be motivated by me. This is important as a good fun, but it also has the character of taking care of your health. … Maybe it would help someone and motivate someone to act when they saw that my health data was improving. (P17, male, 25–34 y/o, CAD, one year or more after diagnosis, but less than two.)

When speaking about social comparison, participants overwhelmingly stated wanting to compare themselves with similar peers, with no particular account other than the similarity itself, occasionally with those who were better off but never worse off. Although notions of similarity could vary slightly from participant to participant, most conceptualized a similar other as someone who, along with the distinctive quality of CVD diagnosis, was in the same age range and of the same gender. This inclination toward demographic similarity might indicate that participants envisioned smaller, highly homophilic communities where each can feel more comfortable, and interactions can, in turn, be more rewarding and effective [29,30] (as cited in [31]). In this regard, one participant commented:

I like sharing personal health data with peers because … other people may have similar experiences so I can fell [*sic*] less alone. I don’t like it when people who have no idea on this subject advise other people or give their opinion. (P5, female, 18–24 y/o, HF, five years or more after diagnosis.)

Other similarity criteria were body weight and disease symptoms, with greater and lesser frequency, respectively.

I like the idea of comparing my data with peers who are similar to me (comparing apples with apples). … The peers whose personal health data interest me are females in the same age bracket and of similar body weight or BMI [body mass index]—not [the] time since diagnosis. It helps to contextualise and compare with people in the same scenario—compare apples with apples and not with pears. (P3)

Age, weight, height and sex for comparison purposes. I would hope such an app would possibly have [the] anonymous messaging capability, to allow for other requests to be made (e.g., “I notice you’ve had a steady decline in your weight over the last 2 months. How have you managed that?”). (P27, male, 65–74 y/o, CAD, five years or more after diagnosis.)

Most participants were inclined to compare themselves with peers of just similar demographic attributes (e.g., “I would be particularly interested in sharing with individuals of a similar demographic as me … I could ’benchmark’ my information [against theirs]”—P29, male, 65–74 y/o, HF, five years or more after diagnosis). Yet a few commented they would do so with “people who have overcome these hard changes” (P22, female, 45–54 y/o, CAD, five years or more after diagnosis). In other words, when upward, the social comparison would be with people “who already have an answer to the problem” (P23, female, 55–64 y/o, CAD, five years or more after diagnosis).

If one considers the limited value some participants attached to peer data (as reported in the first theme), how could social comparison yield benefits at all? One possible answer pertains to the data granularity at which people could compare with similar others. To illustrate, P25 initially hesitated at the utility of (fine-grained) peer data, e.g., heart rate. Later, she stated that contextualizing symptoms, arguably coarse-grained data, might help people realize underdiagnoses.

Perhaps it would be helpful for them [peers] to see in case they too are experiencing similar symptoms and have not yet been diagnosed by a doctor or they have a doctor not taking them seriously and only diagnosing them with anxiety (which [was] what I personally experienced for years until a doctor took me seriously), so maybe seeing and comparing the [symptoms] could help them get diagnosed and get help more quickly. (P25)

Another possible answer to address the potential benefits of social comparison is the driver of comparing oneself with others. Here, we posited that epistemic curiosity [32] could influence participants’ willingness to share personal health data with peers in general and, in particular, compare themselves with similar others. While such curiosity might also be situational, respondents with presumable higher epistemic curiosity seemed to conceptualize this sort of data sharing as an opportunity to learn new ideas that would enhance CVD self-care. Epistemic curiosity has been argued [33] to be related to, but not equivalent to, constructs such as the need for cognition [34] (as cited in [33]), intellectual engagement [35] (as cited in [33]), and openness to experience [36] (as cited in [33]).

[Sharing data with peers] would be good to know what other people in a similar situation are doing [and] how they are being effected [*sic*] by the things they do and monitor. … It is often good to ask and talk to people who have similar problems. Sometimes they can offer good advice which you hadn’t thought of. (P31, male, 75 years or older, HF, five years or more after diagnosis.)

What I like about sharing information with people with this condition is that they can find out how that person is being treated or what follow-up is being given. (P14, male, 18–24 y/o, HF, five years or more after diagnosis.)

I like the thought of having information from others that have heart issues. … I think this info would be valuable and help me with new ideas to better live and treat the disease. (P22)

Not out of enthusiasm for discovering ways to enhance self-care, participants would be less cautious. On the contrary, they demonstrated an awareness of the idiosyncrasies of each condition and of keeping physicians informed before trying something new. Participant accounts also reflected a sense of responsibility to convey this awareness to peers. For example:

While I (we) know not everyone responds to the same treatment, it is nice to know about things I have not yet tried. For example, a lot of people with POTS [postural orthostatic tachycardia syndrome] drink something called liquid IV, I never knew about the product until I saw information about it from my mutuals on Tiktok. I did try it but did not like the taste, so stuck with the propel electrolyte water for my daily hydration needs, which I have shared with other mutuals on private facebook [*sic*] support groups and Tiktok. … I would advise they [peers] ask their doctor first before trying anything whether it be a product or pt [physical therapy] exercise. (P25)

While the detrimental value of peer data and a sense of distrust of similar others were at the opposite end, and close was the thought that such data would make sense only to whom they belong, the positioning that everyone’s experience holds knowledge was at the other end of the spectrum. For example: “[Sharing personal health data] can be helpfull [*sic*] because they [peers] can learn from our own experience” (P10, female, 25–34 y/o, HF, two years or more after diagnosis, but less than five); “Any age group can benefit from a range of experience from a variety of others” (P33, female, 55–64 y/o, HF, five years or more after diagnosis); and “I would feel that everyones [*sic*] experience would hold some type of knowledge” (P22). This experiential knowledge would be especially relevant for the newly diagnosed. “I think knowing what products I use to manage my conditions could be extremely helpful to my peers, especially for those who are newly diagnosed and don’t know about different products that could help them” (P25). Hence, the appraisal of the experience of similar others is a third possible answer to address how social comparison could yield benefits, a question arising from the fact that participants gave limited value to peer data. This approach might be equally applicable to understanding the perceived benefits of sharing personal health data among peers.

Next to social comparison was social support, another prominent affordance of connecting with similar others. By potentially forming smaller, highly homophilic networks, participants pointed out a range of benefits of peer support: from “just talking to” people who “get it”, P24 (male, 18–24 y/o, HF, five years or more after diagnosis) and P25 noted, respectively, to exchanging suggestions, advice, and experiential knowledge, especially relevant in between health-care seeking. As P23 remarked, this support can be emotional and informational: “If I had someone elses [*sic*] data that went through the same thing, it could have helped immensly [*sic*]. It could have relieve [*sic*] my stress levels, and I would have known what to do almost immediately, instead of having to wait to find out from my doctor when I visited her a few weeks later”. In P31’s words, talking about heart disease gains importance in a situation where “it is so easy to become lazy and despondent”. However, though, P23 alluded to the early difficulty in information seeking of finding similar others: “if I knew where to find them [peers]”.

Like her, several participants referred to emotional relief from knowing others (who) are going through similar situations. Thus, hoping to feel emotionally relieved might drive people to connect with peers through data sharing. Additionally, we were inclined to think about emotional relief as an affordance of connecting with similar others. In a similar manner, Genuis and Bronstein [4] positioned this emotional relief as coming from socially constructing a sense of normal. Examples of related remarks include:

I like the idea of sharing this data, because with that and seeing other ppl [*sic*] like me I wouldn’t feel like I’m ’worse’, ’different’ etc. … I feel like reading about other people [*sic*] problems would make me feel more normal, like im [*sic*] not the only one. (P24)

I believe I would have stressed less knowing others were experiencing this [too]. I wouldnt [*sic*] have felt so alone in my fear. (P22)

I think it seems very positive to share, to know and tell the things that happen with me are normal and reassure me or other in similar conditions. (P21, female, 55–64 y/o, CAD, one year or more after diagnosis, but less than two.)

The last driver of connecting with similar others was a sense of altruism. Even though sharing personal health data could not yield benefits for them, some participants remarked they would still do so in the hope of helping someone. For instance:

Overall, I think it can be a good thing to share data with peers. By doing this, I feel that the overall health of people can improve, although it may not make much difference at an individual level, particularly if you are already confident about what weight and BP [blood pressure] should apply to you. (P29)

I would not mind disclosing my personal health data to whom ever [*sic*] wants to know what I have and what I did to help me. If it can help only 1 person, it would be great. (P23)

I would share all my data in hopes to help someone. (P22)

To sum up, we reported that participants conceptualized sharing personal health data with peers as an enabler of connecting with similar others, along with a range of affordances and drivers. Among the affordances, social comparison and social support were prominent. Epistemic curiosity and altruism could instead drive willingness to engage in peer data sharing. Last, we positioned that (feeling) emotional relief might be simultaneously a driver and an affordance of forming smaller, highly homophilic networks through data sharing.

### 3.3. Theme 3: “I Take All Advice from My Peers with a Pinch of Salt”: Or How to Analytically Engage in Data Sharing with Peers

The last theme captured participants’ analytical orientation toward sharing personal health data with peers. The constituent parts of this orientation, or the ways in which research participants expressed themselves about it, suggest that our participants were aware of the difference between holding experiential knowledge and being medically versed and of not treating peer data as a regimen to which to adhere.

The differentiation between people’s experience and medical advice was the first element of the analytical orientation. When asked if they would advise peers, participants often responded they would, limiting themselves strictly to personal experience. For instance: “I will only advise from my personal experience but would encourage my peers to seek medical advice from their doctor” (P32, male, 55–64 y/o, CAD, five years or more after diagnosis); and “If my peers would ask for advice, I would just inform them about my case and not advise them” (P6, female, 45–54 y/o, HF, five years or more after diagnosis). This first constituent part could morph into what P14 called “data as a reference but not as a guideline”.

One size does not fit all. The assertion that what works for one may not work for another was also central to participants’ analytical orientation. For example: “I would advice [*sic*] them if they [peers] asked for help, but I will tell them that [what] helped me … might not be the right thing for them, and that they should seek medical advice as well” (P23); and “Advice from my peers is trustable only to a certain extent because what works for them may not benefit me” (P32).

The other element had to do with double-checking with reliable sources any peer advice or, generally speaking, ideas that seemed to enhance CVD self-care at first glance.

I believe that the information that other people give about their condition could be helpful, however, it would not be reliable for a person to base himself on this, since there has to be a professional in the area. (P14)

I would listen to them and then assess if the advice seems valid, and then I [would] confirm the advice with my dr. or even other peers. (P23)

Even though most participants expressed in one way or another their analytical orientation, a handful of them was less aware, including P18: “[Asking for advice] is not [a] bad idea because it will allow clarification with no confusion. … I do trust the advice my peers give me most of the times”.

### 3.4. Conceptualization(s) Expressed through Rating Activities

Upon understanding participant-defined meaning around the subject of interest, we moved on to quantitative data to analyze how respondents expressed their conceptualization(s) through rating activities. However, before reporting the results, we make it clear how to take them.

When introducing sharing personal health data with peers, we told participants they could choose what items to share, how (e.g., anonymously), and with whom. During rating activities, we portrayed something slightly different, though. We asked participants what pieces of personal (health) data they were likely or unlikely to share but did not make clear if this hypothetical sharing was with the whole community or only with selected members thereof. A handful of them expressed themselves on this point. For instance: “I would be comfortable self-disclosing my personal data only with people I know, or maybe with someone I don’t know, but before, I would like to create a relationship” (P6); and “It would be cool to have [an] option for someone to ’request’ my data when I wouldn’t have the ’everyone’ option. You know, deciding if some stranger can see my data, just to feel [a] complete control” (P24). This inconsistency in survey design limited quantitative data analysis. Therefore, our orientation toward participant ratings was moderate: when participants were likely or unlikely to share any data item, they did so with the whole community.

While it seems reasonable to think that the more sensitive people perceive a data item to be, the less likely they are to share it [8,37], empirical evidence suggests that the expected benefits may outweigh the risks in deciding whether to disclose personal data [38]. Furthermore, people care most about how (others) would use their health information, while the sensitivity of the data would be insignificant [28]. In light of this evidence, we turned our focus to respondents’ reasons for rating a piece of personal health information as likely or unlikely to be shared with peers, rather than ascribing participant choice to the sensitivity of the data. Thus, our account of participant ratings addressed mostly individual data items we could draw a purpose for at the descriptive or interpretative level of the data content.

**Figure 2:**
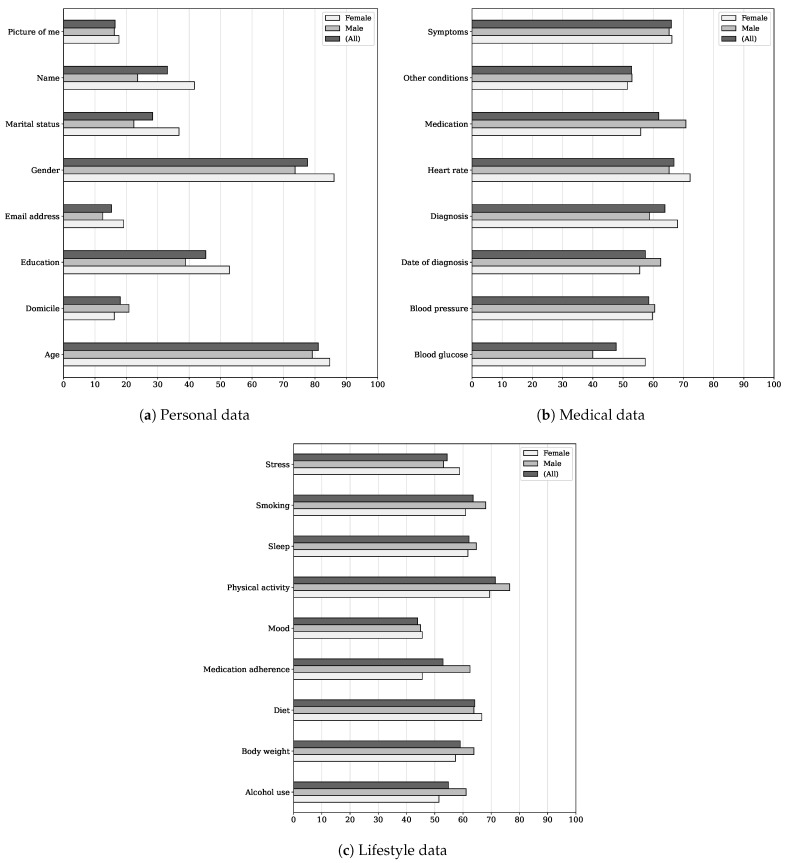
Mean ratings for (**a**) personal, (**b**) medical, and (**c**) lifestyle data items. Here, 0 indicates very unlikely to share, while 100 indicates the exact opposite, i.e., very likely.

Figure 2 depicts gender-disaggregated mean ratings for individual data items. Following an evidence-based lexicon [39] (see also [40]) aimed at bridging the gap between the intended and received meaning of probabilities, we set a rating of 60 as the threshold for saying that participants were likely to share a given piece of personal (health) data. Under this logic, our results show that respondents would choose not to disclose much health information to peers in an eventual data sharing scenario: they were likely to share only 10 out of 25 data items (40%).

Among the data items that participants were likely to share were age and gender. Participants often noted that both types of personal data enable one to relate to another and form smaller, highly homophilic networks, as reported above. But how might it be explained that if a similar other has to have the distinctive quality of CVD diagnosis, the mean rating for this type of medical data was only barely higher than 60 (the threshold) and considerably lower than that for age or gender? One reason is that, in the fictional story with which we introduced the design concept, we told participants that the app made suggestions to follow similar others. Therefore, they could think that sharing their diagnosis was of secondary importance in this context of data sharing. One participant even picked up this app’s social feature and remarked:

The app most propably [*sic*] have [*sic*] a database for all the people using [it], the app itself sort [*sic*] different kinds of stuff together, and decides which group have [*sic*] the same problems. It then suggests that the differrent [*sic*] people could get in touch via the app. If both parties agee [*sic*], the app can put you in contact with each other, or you can chat annonomously [*sic*] with each other. (P23)

It caught our attention that the mean rating for physical activity was relatively high (around 70) as there were few references to this item in participant responses. However, two comments could give a clue to the meaning of sharing this type of lifestyle data. When speaking of people who were similar to them, P3 and P35 (female, 55–64 y/o, HF, five years or more after diagnosis) used the expression “couch potato”. The former to describe herself (“I am a couch potato”; so her peers) and the latter to distance herself from it. “My peers would be people that are active at any age with a pacemaker. … Not a couch potato. … Not the one that sits at home worrying about his heart” (P35). Hence, since physical activity enables people to relate to others, there would be a reason to think it was another similarity criterion shared by many, largely covertly but brought to light through rating activities. The fact that it serves to compare one with another (e.g., Who took more steps yesterday?) could also explain its relatively high rating.

Participants being likely to share heart rate and smoking data also piqued our interest. Firstly, because of the inconsistency between the lesser mutual value that participants attached to sharing this type of medical data and the rating they gave to it. To contextualize and help ourselves explain, we analyzed P25—whose responses portrayed such inconsistency. In a nutshell, she initially stated that data such as heart rate make sense only to whom they belong to or to the individual’s treating physician, as everyone has their baseline for symptoms. Nevertheless, she subsequently answered she would be very likely to share this data item with peers. In elaborating on her choice, P25 expressed that she was very likely to share “symptoms, conditions, [and] treatments [she had tried] both that have and have not worked/helped.” In this context, we posit that sharing one’s heart rate (numerical data) might help make sense of symptoms (categorical data), a level at which it would be easier to relate to and compare with another.

Secondly, it might be the case that participants chose to disclose their smoking status (e.g., never, former, or current) because by doing so, they would learn “techniques to quit smoking” in return (P22), perhaps, however, at the expense of being judged: “I would mind sharing if I drank or smoked. I feel I might judge them as to why they are in the shape they are due to their bad habits” (P35).

As to gender differences in participant ratings, our results show that male participants were likely to share alcohol use, body weight, medication and adherence, and date of diagnosis, in contrast to female ones. Women were likely to self-disclose their diagnosis to the whole community, whereas their counterparts were not. As none of the participants elaborated on the purpose of (not) sharing these data items, we could not explain any gender difference.

On the whole, we found that participants would be likely to share data items provided that items enable one to relate to another and, in turn, social comparison. For example: “Age, smoker or not, diet, use of medicaments, weight, physical activity. I would ask for this data to know if my condition is similiar [*sic*] and how they are trying to attend their disease” (P14); “It would be of interest to me pieces of fata [*sic*] that I can realte [*sic*] to” (P10); “Their blood pressure data and the diet they have chosen to go for to better themselves and if it actually is working for them. That way I get to cross reference with mine” (P13); and “Diagnosis, BP [blood pressure], height, weight, diet, medication, exercise—all for the purpose of comparison with my own situation and for seeking ideas to help improve my own health” (P29). Conversely, participants would be unlikely to share anything that makes them “feel stalk [*sic*] on [*sic*] every aspect of life” (P7) or, generally speaking, is irrelevant to the context of data sharing. Hence, we can argue that a sense of pragmatism led participants to choose what pieces of personal health data they were likely to share with peers.

Mean ratings suggested that participants were unlikely to share most pieces of their personal (health) data. However, we could not capture any potential variance in sharing preference potentially attributable to the granularity or data level of detail. Nor how different it would be to share if there was a link (or relationship) among peers. Even so, we propose enabling people to choose what to share in each situation individually. For the rest, it still is open how to gauge participant conceptualization taking into account data granularity and adaptable data-sharing preferences.

## 4. Discussion

### 4.1. Principal Findings

We designed a survey to gather attitudes toward sharing personal health data with peers in people who reported they had been diagnosed with CVD. Following the reflexive thematic analysis method, we identified and developed three patterns of meaning (themes). First, our findings revealed that participants formed their opinion on peer data sharing by situating it relative to existing sources of self-care support or by weighing potential, mainly disadvantageous outcomes. Accordingly, we found that for some, interacting with peers in such a manner yields uncertain benefits and is unnecessary, especially when there is a high sense of self-efficacy. Not knowing how to use peer data or how practical it would be to learn about each other’s “numbers” contributed to participants’ feelings of uncertainty. In relation to this point, participants added that peer data make sense only to whom the data belong or to the individual’s treating physician. Another factor that contributed to feeling uncertain about (the benefits of) data sharing was the utility of personal health data in the hands of peers. “Who knows if peers are medically versed enough to understand these data?”

Second, we narrated that while the lesser or even detrimental value of peer data and a sense of distrust of similar others were at one end of the spectrum, the position that everyone’s experience holds knowledge was at the opposite end. This positioning led participants to the notion that sharing personal health data with peers constitutes an opportunity to learn new ideas that would enhance CVD self-care, primarily through social comparison. Most participants were inclined to compare themselves with peers of just similar demographic attributes, particularly age and gender. Still, a few remarked they would do so with those “who already have an answer to the problem”. However, to share data in general and, in particular, compare themselves with others, people first have to connect with those facing similar health issues. Hence, in a context where people need to address how to find similar others—especially since the experience of others often shapes individuals’ notions of normal [4]—data sharing could serve to connect sufferers with one another.

Third, we broke down the analytical orientation participants adopted toward sharing personal health data with peers. In a nutshell, respondents differentiated between holding experiential knowledge and being medically versed. Next was the assertion that what works for one may not work for another. The other element of the orientation had to do with double-checking with reliable sources any peer advice or, in general, ideas that seemed to enhance CVD self-care.

Our quantitative data analysis showed that participants were likely to share 10 types of personal health data with peers, including age, gender, diagnosis, heart rate, medication, symptoms, and physical activity. However, in perspective, we found that respondents would choose not to share much information about themselves, since the 10 data items represented 40% of the 25 we asked them to rate. As to the purpose, they would be likely to share data items provided that items enable one to relate to another and, in turn, social comparison. Conversely, they would be unlikely to share anything irrelevant to the context of data sharing. For the rest, we did not capture any variance in sharing preference potentially attributable to data granularity or adaptable data-sharing settings.

### 4.2. Comparison with Prior Work

The body of literature dealing with the sharing of personal health data among peers is limited, and so is our understanding [5,8]. The nature and purpose of this sharing, or what it involves and what it does not, are still blurred. It is perhaps for this reason that we found that the literature used the term itself to refer to a range of similar activities [10]. To illustrate, when the sharing is one-way, it could denote disclosure or even donation. In this study, participants were sometimes reluctant to interact with peers through data sharing since they did not know how others would use or benefit from their data. Therefore, it seems plausible to think they conceptualized the subject of interest unidirectionally.

When the sharing is two-way, it could denote an exchange of assets. Bussone et al. [8] positioned it in such a manner. “Sharing data is best thought of as an exchange between individual community members, based around a common purpose, such as a question like how to better self-manage health” (p. 17). Like them, we consider that the sharing of personal health data among peers might prove beneficial for chronic disease self-care by serving as a means of developing a shared understanding of health-related issues. However, our notion is slightly different from that of Bussone and colleagues. We are inclined to think that data sharing can take several forms. More specifically, it can be one- or two-way according to the purpose it serves within the overarching one. To help ourselves explain our notion, we borrow inspiration from the journey mapping concept.

The literature has reported how people dealing with life-disrupting, health-related challenges expend great effort in information-seeking behavior to validate personal experiences [2,4]. Since the experience of fellow sufferers is a source of information that people often seek, they need to address how to find peers early in the journey. In this context, we position that one-way data-sharing serves to connect sufferers with one another. Here health technology could make recommendations of similar others, considering criteria by which people relate most to each other, e.g., age and gender, or other adaptable preferences. That is, it would play an intermediary role, though with limitations. To illustrate, the effectiveness of intermediating among peers would be contingent upon the number and variety of participating people. Nonetheless, we think that a potential line of future work is how to design to facilitate locating others with relevant experiences and lowering the threshold of sharing these. For example, sharing experiences can be decoupled from sharing physiological data.

Upon helping connect one with another, a prominent affordance of data sharing, as expressed by participants, is that of social comparison. Our findings suggested that epistemic curiosity could lead people to compare themselves with peers, as participants recognized in mutual data sharing an opportunity to learn ideas that would enhance CVD self-care. Hence, another line of future work is how to design for social comparison enabled by peer data sharing so that the comparison is conducive to gaining insights into new ways of enhancing self-care.

As to the contributions of this research to the literature, we found that feeling that data sharing yields uncertain benefits is a barrier to interacting with peers in such a manner. While a recent narrative review [10] identified trust, identity, privacy, and security concerns as barriers to the sharing of personal health data across multiple settings, including but not limited to peers, it assumed that data sharing is beneficial per se. On the contrary, our findings showed that participants were far from taking this for granted.

When taking together all the rich and complex participant accounts, this study offered a new perspective on the sharing of personal health data among peers. This perspective is especially relevant in a context where the literature is scarce in general and, in particular, in qualitative research [10]. To illustrate, studies such as Frost et al. [6] and Vaala et al. [5], the former not included in the narrative review in contrast to the latter, have reported, albeit sometimes inconsistently, what health information people with cancer and type 1 diabetes mellitus, respectively, were likely to share with peers. They also reported what factors influence people’s willingness to share, in what direction, and to what extent. Our study adds to these works by elaborating on how people with CVD conceptualize peer data sharing, thus extending to a different population/pathology and taking a qualitative orientation to unpack the concept from the participant’s perspective.

To our knowledge, Bussone et al. [8] is the only earlier study that, like ours, investigates the sharing of personal health data among peers. Theirs addresses a different population, namely people with HIV interested in interacting with peers in such a manner. By exploring informants’ meanings of trust, identity, privacy, and security in sharing data with peers, and what considerations they made to engage in this sharing, Bussone and colleagues drew design recommendations to foster responsible data sharing. Among the recommendations were giving participants complete control over what to share and for how long, ensuring that community members meet eligibility criteria, and encouraging community norms such as mutual respect and appropriate data sharing, to name a few. While these design recommendations remain relevant, we find that embracing the voices of the “non-aligned” rather than discouraging health technology design made it more robust, thus providing a critical perspective that even challenged our beliefs. Besides that, our study examines the notion of sharing with more nuance, particularly by distinguishing between disclosing one’s and exploring others’ data.

### 4.3. Limitations and Strengths

After reporting and contextualizing the findings, we make it clear how to take them, though not extensively in the first place. A limitation of the self-selection recruitment strategy is that we cannot be sure that respondents correctly reported their match to the eligibility criteria. Future studies with direct recruitment from a known patient population might help overcome this limitation.

Along with the fact that we turned to a proxy population, there is reason to believe that the study sample consisted of people with Internet literacy higher than the average. Among the skills this literacy refers to is adopting protective measures against, for example, inappropriate disclosure of personal or sensitive information (privacy risks) and unauthorized third-party access to one’s digital assets (security risks) [41]. In this study, some participants stated that they would not engage in peer data sharing, even despite its benefits, since personal health data would never be secure in the digital realm.

On the other hand, we drew upon the survey method’s practicalities of online delivery and self-administration. Therefore, a salient strength of our study was reaching out to a geographically dispersed population, thereby hearing diverse voices and potentially enriching our understanding of the subject of interest. At the same time, we acknowledge a lack of cultural variation since all participants were from the Western Hemisphere and none, for example, from the Far East.

## 5. Conclusions

We look forward to collaborative health technology built on data sharing among peers. As people with CVD are to engage in self-monitoring routinely and are to (gain knowledge to) be aware of worsening disease symptoms and the corresponding course of action, our positioning is that this sharing might serve as a means of developing a shared understanding of health-related issues. However, when situating the research findings relative to the existing literature, we found that both designers and end-users lacked, in turn, a shared understanding of the subject of interest. To illustrate, we showed that participants sometimes expressed uncertain benefits of interacting with peers in such a manner, in contrast to a recent narrative review that assumed data sharing is beneficial per se. Moreover, we suggested that peer data sharing can take several forms and thus serve different purposes, from connecting people with others in similar positions to helping them to learn new ideas that would enhance CVD self-care. Overall, the rich and complex participant accounts presented herein offered a new perspective on how people with CVD conceptualized disclosing personal health data to peers and exploring those of others facing similar health challenges.

## Data Availability

The quantitative and qualitative datasets this paper analyzes are not made publicly available.

## References

[1] Riegel B., Moser D.K., Buck H.G., Dickson V.V., Dunbar S.B., Lee C.S., Lennie T.A., Lindenfeld J., Mitchell J.E., Treat-Jacobson D.J. (2017). Self-care for the prevention and management of cardiovascular disease and stroke: A scientific statement for healthcare professionals from the American Heart Association. J. Am. Heart Assoc..

[2] O’Kane A.A., Park S.Y., Mentis H., Blandford A., Chen Y. (2016). Turning to peers: Integrating understanding of the self, the condition, and others’ experiences in making sense of complex chronic conditions. Comput. Support. Coop. Work (CSCW).

[3] Penrod J., Hupcey J.E., Shipley P.Z., Loeb S.J., Baney B. (2012). A model of caregiving through the end of life: Seeking normal. West. J. Nurs. Res..

[4] Genuis S.K., Bronstein J. (2017). Looking for “normal”: Sense making in the context of health disruption. J. Assoc. Inf. Sci. Technol..

[5] Vaala S.E., Lee J.M., Hood K.K., Mulvaney S.A. (2018). Sharing and helping: Predictors of adolescents’ willingness to share diabetes personal health information with peers. J. Am. Med. Inform. Assoc..

[6] Frost J., Vermeulen I.E., Beekers N. (2014). Anonymity versus privacy: Selective information sharing in online cancer communities. J. Med. Internet Res..

[7] Zhang X., Liu S., Chen X., Wang L., Gao B., Zhu Q. (2018). Health information privacy concerns, antecedents, and information disclosure intention in online health communities. Inf. Manag..

[8] Bussone A., Kasadha B., Stumpf S., Durrant A.C., Tariq S., Gibbs J., Lloyd K.C., Bird J. (2020). Trust, identity, privacy, and security considerations for designing a peer data sharing platform between people living with HIV. Proc. ACM Hum.-Comput. Interact..

[9] Zhu H., Colgan J., Reddy M., Choe E.K. (2016). Sharing patient-generated data in clinical practices: An interview study. AMIA Annu. Symp. Proc..

[10] Simpson E., Brown R., Sillence E., Coventry L., Lloyd K., Gibbs J., Tariq S., Durrant A.C. (2021). Understanding the Barriers and Facilitators to Sharing Patient-Generated Health Data Using Digital Technology for People Living with Long-Term Health Conditions: A Narrative Review. Front. Public Health.

[11] Creswell J.W., Clark V.L.P. (2017). Designing and Conducting Mixed Methods Research.

[12] Braun V., Clarke V., Boulton E., Davey L., McEvoy C. (2021). The online survey as a qualitative research tool. Int. J. Soc. Res. Methodol..

[13] Braun V., Clarke V. (2013). Successful Qualitative Research: A Practical Guide for Beginners.

[14] Willig C. (2013). Introducing Qualitative Research in Psychology.

[15] Eyal P., David R., Andrew G., Zak E., Ekaterina D. (2021). Data quality of platforms and panels for online behavioral research. Behav. Res. Methods.

[16] Chandler J.J., Paolacci G. (2017). Lie for a dime: When most prescreening responses are honest but most study participants are impostors. Soc. Psychol. Personal. Sci..

[17] Buskermolen D.O., Terken J. Co-constructing stories: A participatory design technique to elicit in-depth user feedback and suggestions about design concepts. Proceedings of the 12th Participatory Design Conference: Exploratory Papers, Workshop Descriptions, Industry Cases-Volume 2.

[18] Mamykina L., Smaldone A.M., Bakken S.R. (2015). Adopting the sensemaking perspective for chronic disease self-management. J. Biomed. Inform..

[19] Sherwin K. (2018). Card Sorting: Uncover Users’ Mental Models for Better Information Architecture. https://www.nngroup.com/articles/card-sorting-definition/.

[20] Braun V., Clarke V. (2006). Using thematic analysis in psychology. Qual. Res. Psychol..

[21] Clarke V., Braun V., Teo T. (2014). Thematic analysis. Encyclopedia of Critical Psychology.

[22] Byrne D. (2021). A worked example of Braun and Clarke’s approach to reflexive thematic analysis. Qual. Quant..

[23] Reicher S. (2000). Against methodolatry: Some comments on Elliott, Fischer, and Rennie. Br. J. Clin. Psychol..

[24] Braun V., Clarke V. (2021). One size fits all? What counts as quality practice in (reflexive) thematic analysis?. Qual. Res. Psychol..

[25] Dinev T., Hart P. (2006). An extended privacy calculus model for e-commerce transactions. Inf. Syst. Res..

[26] Bandura A. (1977). Self-efficacy: Toward a unifying theory of behavioral change. Psychol. Rev..

[27] Warner L.M., French D.P., Hagger M.S., Cameron L.D., Hamilton K., Hankonen N., Lintunen T. (2020). Self-Efficacy Interventions. The Handbook of Behavior Change.

[28] Krahe M., Milligan E., Reilly S. (2019). Personal health information in research: Perceived risk, trustworthiness and opinions from patients attending a tertiary healthcare facility. J. Biomed. Inform..

[29] Cornwell B., Schafer M.H., George L.K., Ferraro K.F. (2016). Social networks in later life. Handbook of Aging and the Social Sciences.

[30] Ma L., Krishnan R., Montgomery A.L. (2015). Latent homophily or social influence? An empirical analysis of purchase within a social network. Manag. Sci..

[31] Qiao W., Yan Z., Wang X. (2021). Join or not: The impact of physicians’ group joining behavior on their online demand and reputation in online health communities. Inf. Process. Manag..

[32] Litman J.A., Seel N.M. (2012). Epistemic curiosity. Encyclopedia of the Sciences of Learning.

[33] Mussel P. (2010). Epistemic curiosity and related constructs: Lacking evidence of discriminant validity. Personal. Individ. Differ..

[34] Cacioppo J.T., Petty R.E. (1982). The need for cognition. J. Personal. Soc. Psychol..

[35] Goff M., Ackerman P.L. (1992). Personality-intelligence relations: Assessment of typical intellectual engagement. J. Educ. Psychol..

[36] Digman J.M. (1990). Personality structure: Emergence of the five-factor model. Annu. Rev. Psychol..

[37] Ma X., Hancock J., Naaman M. Anonymity, intimacy and self-disclosure in social media. Proceedings of the 2016 CHI conference on Human Factors in Computing Systems.

[38] Trepte S., Scharkow M., Dienlin T. (2020). The privacy calculus contextualized: The influence of affordances. Comput. Hum. Behav..

[39] Wintle B.C., Fraser H., Wills B.C., Nicholson A.E., Fidler F. (2019). Verbal probabilities: Very likely to be somewhat more confusing than numbers. PLoS ONE.

[40] Ho E.H., Budescu D.V., Dhami M.K., Mandel D.R. (2015). Improving the communication of uncertainty in climate science and intelligence analysis. Behav. Sci. Policy.

[41] Saito N. (2015). Internet Literacy in Japan. https://www.oecd-ilibrary.org/science-and-technology/internet-literacy-in-japan_5js0cqpxr6bq-en.

